# Genetic differentiation and conservation insights into *Salicornia iranica* subsp. *sinus-persica* from Musa Bay using SCoT markers and DNA barcodes

**DOI:** 10.1016/j.jgeb.2025.100563

**Published:** 2025-09-03

**Authors:** Fatemeh Nasernakhaei, Mahyar Zahraei

**Affiliations:** a Department of Plant Production and Genetics, Faculty of Agriculture, Shahid Chamran University of Ahvaz, Ahvaz Postal Code: 61357-43311, Iran; b Department of Biology, Faculty of Science, Shahid Chamran University of Ahvaz, Ahvaz, Iran

**Keywords:** Molecular profiling, ITS, *trnH-psbA*, Halophytes, Intraspecific variation, Phylogenetic relationships, Conservation genetics

## Abstract

Understanding genetic diversity is essential for the conservation and management of halophyte species, especially in ecologically sensitive and industrially impacted regions. In this study, 52 accessions of *Salicornia iranica* subsp. *sinus-persica* were collected from diverse microhabitats across Musa Bay, including coastal zones, islands, and petrochemical sites. Following comprehensive morphological assessments showing high phenotypic similarity, and to ensure broad ecological coverage, 16 accessions representing all major microhabitats were selected for molecular analysis. SCoT markers and DNA barcoding (ITS and *trnH-psbA* regions) were employed to investigate their genetic structure. The SCoT markers revealed considerable polymorphism and partial genetic differentiation, especially between accessions from petrochemical and natural saline habitats. ITS sequencing identified five haplotypes without forming a monophyletic clade, indicating shallow divergence and possible ancestral polymorphism. In contrast, no variation was detected in the plastid region *trnH-psbA*. These results represent the first global report of ITS and *trnH-psbA* haplotypes for this subspecies. While the findings are preliminary due to limited sample size and marker coverage, they provide a critical baseline for future studies employing high-resolution genomic tools. Our findings underscore the influence of habitat fragmentation and anthropogenic pressures on genetic patterns and emphasize the importance of conserving genetically distinct accessions in ecologically vulnerable habitats to support the long-term survival and sustainable utilization of this coastal halophyte.

## Highlights

• First molecular study of *S. iranica* subsp*. sinus-persica* using SCoT and barcoding.
• SCoT markers reveal genetic structuring across coastal and industrial habitats.
• ITS haplotypes show site-specific patterns and localized divergence.
• Phylogeny indicates shallow divergence and ancestral polymorphism.
• Novel ITS and *trnH-psbA* haplotypes identified in this native halophyte.

## 1 Introduction

**Fig. 1:**
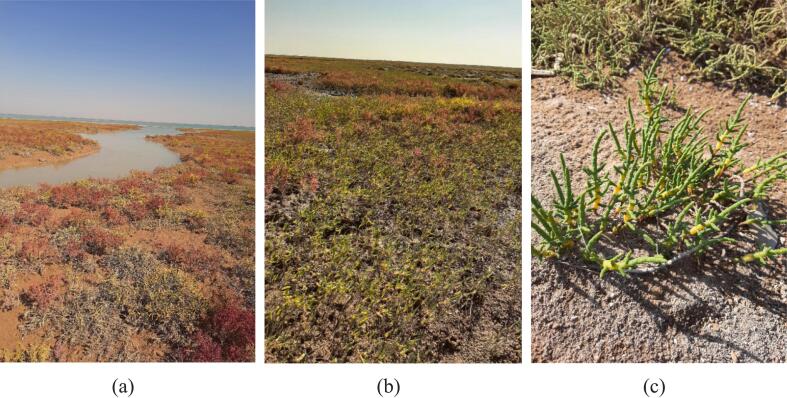
*Salicornia iranica* subsp. *sinus-persica* in the Musa Bay area: (a, b) representative habitat views; (c) close-up of the plant.

*Salicornia iranica* subsp. *sinus-persica* (Akhani) Chatren. & Akhani (Chenopodiaceae) (Fig. 1) is a narrowly distributed halophytic taxon native to the southern coasts of Iran, including Musa Bay. This taxon was initially described as *Salicornia sinus-persica*^1^ but was later reclassified as a subspecies of *S. iranica* Akhani based on integrated morphological and molecular evidence. This updated taxonomy reflects its close affinity with other Iranian *Salicornia* taxa while recognizing its distinct diploid lineage (2n = 18) and ecological specificity.^2^

Taxonomic classification within *Salicornia* has long been challenging due to high morphological plasticity, polyploidy, and frequent interspecific hybridization.^3^ These complexities often lead to misidentification and ambiguous species boundaries.^2^ To overcome these challenges, recent studies emphasize the importance of integrative approaches that combine morphological, ecological, and molecular data.^4^ Among molecular tools, the nuclear internal transcribed spacer (ITS) and the plastid *trnH-psbA* intergenic spacer markers have shown promise for species-level identification in *Salicornia*, though their effectiveness varies depending on data quality and analytical methods.^5^ However, the genetic diversity of *S. iranica* subsp. *sinus-persica* remains unexplored.

Beyond its taxonomic relevance, *S. iranica* subsp. *sinus-persica* holds notable ecological and economic value. It generates substantial biomass under saline irrigation and has been proposed as a potential forage crop in seawater-based cultivation systems.^6^ Although our study did not assess forage traits directly, it offers foundational genetic insights that can guide future efforts in selecting genotypes with enhanced biomass productivity and stress resilience. Given its proposed role in seawater-based forage systems, understanding the genetic variation of *S. iranica* subsp*. sinus-persica* represents a critical initial step in evaluating its potential as a sustainable feed resource.

Given the ecological challenges in arid and salt-affected coastal regions, investigating its genetic structure through molecular tools such as start codon targeted (SCoT) markers^7^, ^8^ and DNA barcodes^9^, ^10^ may offer insights into mechanisms of local adaptation and inform future utilization strategies.

This study aimed to evaluate the genetic diversity and phylogenetic relationships of *S. iranica* subsp. *sinus-persica* accessions from Musa Bay using SCoT markers and ITS-based DNA barcoding. Additionally, the plastid region *trnH-psbA* was analyzed to assess haplotype variation. The findings provide novel data on the genetic structure of this subspecies and serve as a foundation for developing targeted conservation measures to preserve halophytic biodiversity in coastal ecosystems.

## 2 Materials and methods

### 2.1 Plant material

**Fig. 2:**
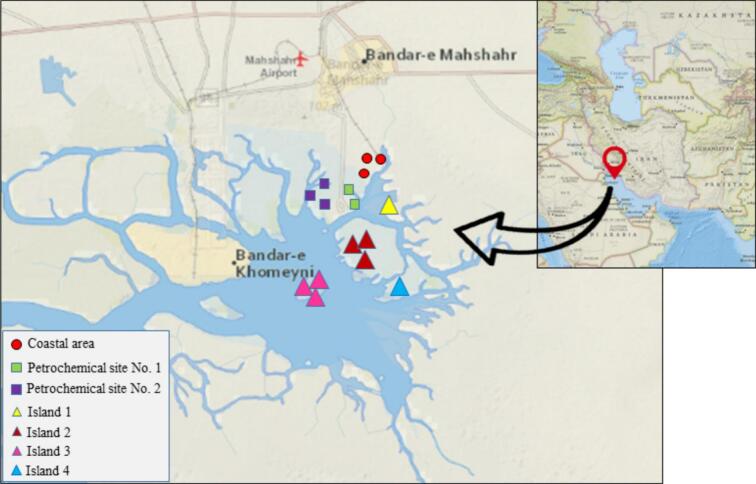
Distribution of *Salicornia iranica* subsp. *sinus-persica* accessions in Musa Bay using data from the National Geologic Map Database (NGMDB).

A total of 52 accessions of *S. iranica* subsp. *sinus-persica* were collected from various habitats across the Musa Bay area, including islands, coastal zones, and two petrochemical sites. Specimens were morphologically identified using the diagnostic key provided by Chatrenoor and Akhani (2021). Following a comprehensive morphological assessment that revealed high phenotypic uniformity, 16 representative accessions (Fig. 2, Table 1) were selected based on three criteria: (i) ecological coverage of all microhabitats, (ii) geographical representation across environmental gradients, and (iii) quality of extracted DNA. Tissue samples from all accessions were preserved in silica gel for molecular analysis. High-resolution field photographs of live specimens in their natural environment were taken, and detailed observations were made using an Olympus-SZX12 stereomicroscope. This combined documentation, together with the silica gel-preserved tissues, serves as voucher material deposited in the Herbarium of Shahid Chamran University of Ahvaz (Department of Plant Production and Genetics).

**Table 1:** Geographic locations of the studied *Salicornia iranica* subsp. *sinus-persica* accessions in Musa Bay.

**Accession code**	**Collection site**	**Longitude (E)**	**Latitude (N)**
Sal-1	Coastal area	49°11′21.4″	30°29′33.3″
Sal-2	Coastal area	49°11′21.4″	30°29′25.2″
Sal-3	Coastal area	49°11′20.4″	30°29′12.2″
Sal-4	Petrochemical site No. 1	49°10′53.2″	30°28′35.0″
Sal-5	Petrochemical site No. 1	49°10′53.8″	30°28′34.3″
Sal-6	Petrochemical site No. 2	49°10′28.9″	30°28′31.9″
Sal-7	Petrochemical site No. 2	49°10′28.7″	30°28′31.8″
Sal-8	Petrochemical site No. 2	49°10′28.9″	30°28′31.9″
Sal-9	Island 1	49°12′40.2″	30°28′21.0″
Sal-10	Island 3	49°09′45.3″	30°25′09.6″
Sal-11	Island 3	49°09′45.5″	30°25′09.7″
Sal-12	Island 3	49°09′45.7″	30°25′09.9″
Sal-13	Island 2	49°11′11.4″	30°25′41.8″
Sal-14	Island 2	49°11′11.5″	30°25′42.7″
Sal-15	Island 2	49°11′12.3″	30°25′45.8″
Sal-16	Island 4	49°13′28.4″	30°25′15.9″

Genomic DNA was extracted from 20 mg of silica-dried tissue using the AnaCell Plant DNA Extraction Kit (Iran), following the manufacturer’s instructions. DNA quality and quantity were evaluated using 0.8 % agarose gel electrophoresis and spectrophotometry.

### 2.2 SCoT PCR amplification

SCoT amplification was performed using eight primers selected from an initial screening of twelve primers described by Collard and Mackill (2009). Only primers producing reproducible and scorable banding patterns were utilized. Each 25 μL PCR reaction contained 12.5 μL of 2X Master Mix (Ampliqon), 1 μL of primer (10 pmol/μL), 1 μL of genomic DNA (30–40 ng), and 10.5 μL of double-distilled water. The PCR cycling conditions comprised an initial denaturation step at 94 °C for 2 min, followed by 35 cycles of denaturation at 94 °C for 50 s, primer annealing at 50 °C for 45 s, and extension at 72 °C for 2 min, with a final extension at 72 °C for 10 min. The amplified products were electrophoresed on 1.5 % agarose gel and visualized using YTA Safe Stain under UV illumination.

### 2.3 DNA barcoding and sequencing

The internal transcribed spacer (ITS) region was amplified using ITS1^11^ 5′-AGAAGTCGTAACAAGGTTTCCGTAGG-3′ and ITS4^12^ 5′-TCCTCCGCTTATTGATATGC-3′. The *trnH-psbA* intergenic spacer was amplified with the primers psbA^13^ 5′-GTTATGCATGAACGTAATGCTC-3′ and trnH2^14^ 5′-CGCGCATGGTGGATTCACAATCC-3′. The PCR protocol for ITS amplification began with an initial denaturation step at 94 °C for 3 min, followed by 30 cycles consisting of denaturation at 94 °C for 30 s, primer annealing at 52 °C for 30 s, and extension at 72 °C for 1 min, with a final extension at 72 °C for 10 min. For *trnH-psbA* amplification, the protocol was identical, except that the annealing temperature was adjusted to 53 °C, 40 cycles were performed, and a final extension step of 2 min was used. The amplified PCR products were purified and then sequenced in both directions using an ABI 3730xl DNA Analyzer, using the same primers as those employed for PCR amplification.

### 2.4 Data analysis

For SCoT markers, amplified bands were scored as present (1) or absent (0) using CLIQS Gel Image Analysis Software (TotalLab, UK). Each polymorphic band was considered an independent locus. To evaluate the efficiency of the primers, the number of polymorphic bands (NP), polymorphism information content (PIC),^15^ and marker index (MI)^16^ were calculated. Genetic similarity among accessions was calculated using the Jaccard coefficient in NTSYS-PC^17^ version 2.01. Cluster analysis of the SCoT profiles was conducted using the unweighted pair group method with arithmetic mean (UPGMA). Principal coordinates analysis (PCoA) was also performed to visualize genetic relationships among accessions and to validate clustering patterns.

ITS and *trnH-psbA* sequences were aligned using BioEdit (version 7).^18^ Sequence variation and haplotype diversity were analyzed using DnaSP (version 6),^19^ and haplotype networks were constructed with PopART (version 1.7). Phylogenetic reconstruction was conducted exclusively using ITS haplotypes derived from all 16 accessions (Table 1), as ITS is widely applied for inferring relationships among closely related taxa. A neighbor-joining (NJ) tree was generated in MEGA (version 11.0.13)^20^ with 1000 bootstrap replicates. Additional ITS sequences showing ≥ 95 % identity were retrieved from GenBank to contextualize phylogenetic placement. Although the *trnH-psbA* region was successfully sequenced in 10 accessions, it was excluded from phylogenetic analysis due to a lack of sequence variation and limited sampling, ensuring that tree reconstruction was based solely on the more informative ITS dataset.

## 3 Results

### 3.1 Polymorphism of amplified products

Eight SCoT primers were used, generating 80 clear and reproducible bands, of which 66 (82.5 %) were polymorphic. The number of bands per primer ranged from 3 (SCoT34) to 18 (SCoT1). The highest polymorphism (100 %) was recorded for SCoT3, SCoT20, and SCoT34, whereas SCoT11 showed the lowest polymorphism (47 %). The polymorphism information content (PIC) ranged from 0.14 (SCoT11) to 0.45 (SCoT34), with an average of 0.27. The mean marker index (MI) was calculated to be 1.91, indicating the effectiveness of the SCoT primers in detecting genetic variation among the studied accessions (Table 2).

**Table 2:** SCoT markers and parameter calculation.

**Primer**	**Primers sequences 5′→3′**	**GC%**	**NB**	**NP**	**P%**	**PIC**	**MI**	**SB (bp)**
SCoT1	CAACAATGGCTACCACCA	50	18	17	94	0.18	3.01	250–2800
SCoT3	CAACAATGGCTACCACCG	50	5	5	100	0.3	1.53	750–1600
SCoT5	CAACAATGGCTACCACGA	50	6	5	83	0.25	1.06	800–3000
SCoT11	AAGCAATGGCTACCACCA	50	17	8	47	0.14	0.53	280–3000
SCoT14	ACGACATGGCGACCACGC	67	13	11	84	0.32	3.07	200–3000
SCoT19	ACCATGGCTACCACCGGC	67	11	10	90	0.33	3.03	500–3000
SCoT20	ACCATGGCTACCACCGCG	67	7	7	100	0.24	1.71	800–3000
SCoT34	ACCATGGCTACCACCGCA	61	3	3	100	0.45	1.36	800–1700
			80	66		0.27	1.91	

Total Number of Bands (NB), Number of Polymorphic Bands (NP), Polymorphic Information Content (PIC), Marker Index (MI), and Size of Bands (SB)

### 3.2 Cluster and principal coordinates analysis based on SCoT markers

**Fig. 3:**
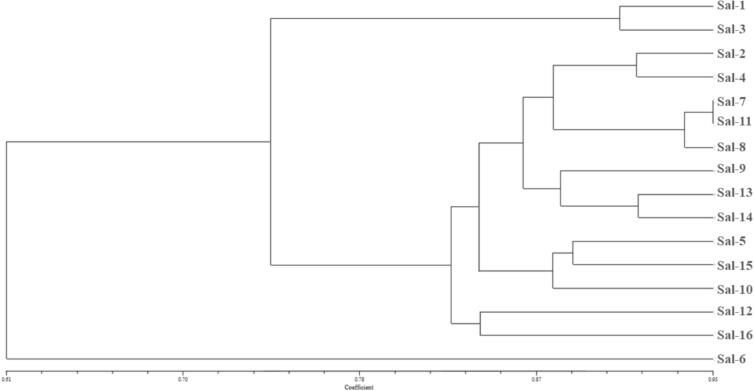
UPGMA cluster analysis of *Salicornia iranica* subsp. *sinus*-*persica* accessions based on SCoT markers using the Jaccard similarity coefficient.

Cluster analysis based on the Jaccard coefficient and UPGMA method grouped the 16 accessions into two main clusters (Fig. 3), which were distinguished based on the highest pairwise genetic distances, indicating clear genome-wide divergence between the groups. Sal-6 (Petrochemical site No. 2) formed a distinct branch, while Sal-1 and Sal-3 (coastal area) clustered together. The remaining accessions formed subclusters that partially followed their geographic origins, with island accessions showing more coherence and coastal and petrochemical accessions displaying overlap across groups. This partial clustering pattern reflects limited congruence between genetic and geographic distances.

**Fig. 4:**
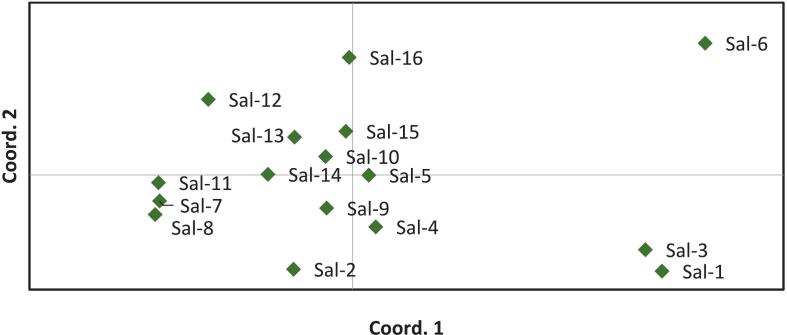
Principal coordinates analysis (PCoA) of studied *Salicornia iranica* subsp. *sinus*-*persica* accessions based on SCoT markers.

Principal coordinates analysis (PCoA) supported these clustering results and visualized the genetic relationships among accessions (Fig. 4). It also showed the intermixing of accessions across the coordinates. The first three principal coordinates accounted for 50.63 % of the total variation.

### 3.3 DNA barcoding analysis

**Fig. 5:**
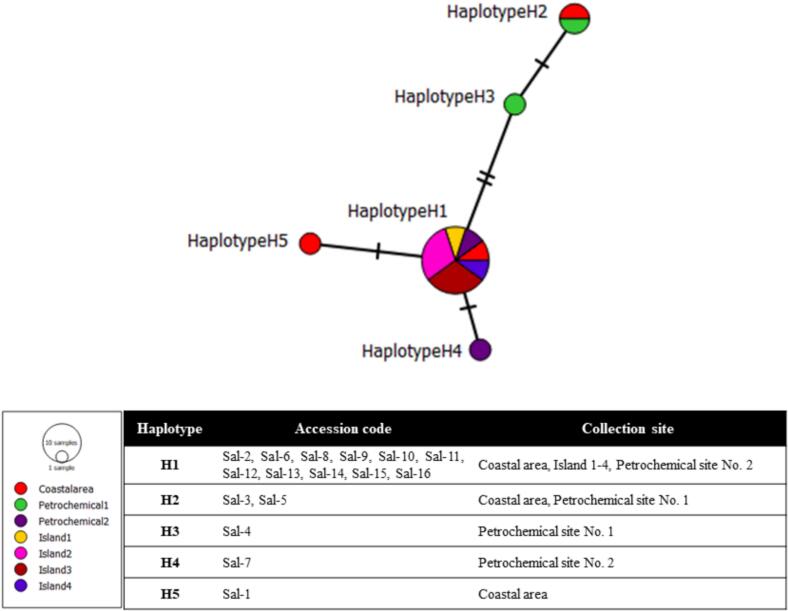
Distribution and collection site of ITS haplotypes among *Salicornia iranica* subsp. *sinus-persica* accessions.

The aligned ITS region (548 bp) revealed five haplotypes (H1–H5) among the 16 accessions of *S. iranica* subsp. *sinus-persica*, with haplotype H1 being the most frequent in 11 accessions (Fig. 5). Eight polymorphic sites were identified, including three singleton variable sites (positions 72, 185, and 399) and two parsimony-informative sites (positions 106 and 115). Notably, haplotype H5 exhibited unique substitutions (399A, 403G, and 404A) and lacked the deletions observed in the other haplotypes (Table 3). The haplotype diversity (Hd) was 0.507.

**Table 3:** Variable nucleotide positions in the ITS region among *S. iranica* subsp. *sinus-persica* accessions. Numbers indicate nucleotide positions within the aligned sequence. Dashes (−) represent gap.

	**ITS1**					**ITS2**		
**Position**	**72**	**106**	**115**	**185**		**399**	**403**	**404**
**Haplotypes**								
H1	C	C	A	C		G	−	−
H2	T	T	C	C		G	−	−
H3	C	T	C	C		G	−	−
H4	C	C	A	T		G	−	−
H5	C	C	A	C		A	G	A

BLAST comparisons using the NCBI database indicated that H1 shared the highest sequence identity with *S. persica* (EF453460.1). Haplotypes H2 and H3 showed 99.31 % and 99.61 % identity to the same reference, respectively. In contrast, H4 and H5 exhibited closer similarity to *S. europaea* (AB537514.1), with 99.83 % and 99.66 % identity, respectively. All ITS sequences generated in this study were submitted to GenBank under accession numbers PV392200–PV392215.

Due to limitations in DNA quantity, the *trnH-psbA* plastid region was sequenced in only 10 accessions, resulting in an aligned length of 525 bp. No nucleotide variation or haplotype differentiation was observed among these sequences. BLAST analysis revealed 100 % identity and 98 % query coverage with *S. persica* (OM397216). These sequences were deposited in GenBank under accession numbers OR234357–OR234366. To the best of our knowledge, this constitutes the first report of *trnH-psbA* sequences for *S. iranica* subsp. *sinus-persica*.

### 3.4 Phylogenetic analysis of ITS haplotypes

**Fig. 6:**
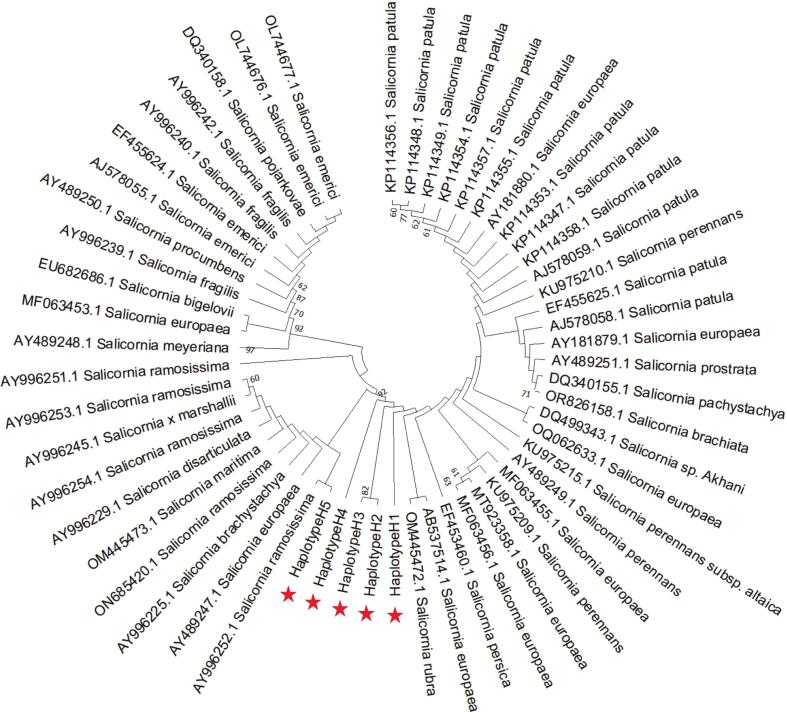
Neighbor-joining phylogenetic tree of *Salicornia* species based on nuclear ITS sequence identity, showing haplotypes of *S. iranica* subsp. *sinus-persica* and related taxa. Red stars indicate the ITS haplotypes newly identified in this study. Bootstrap support values (>60 %) are shown at the nodes.

A neighbor-joining (NJ) phylogenetic tree was constructed using the five ITS haplotypes (H1–H5) identified in this study, along with ITS sequences of related *Salicornia* species retrieved from GenBank (Table S1). Only sequences with ≥ 95 % identity to the Musa Bay accessions were included. Phylogenetic analysis revealed that the five ITS haplotypes of *S. iranica* subsp. *sinus-persica* did not form a single distinct clade. Instead, they appeared in a stepwise pattern across the tree, with H5 clustering closer to *S. ramosissima*, while others were gradually connected to lineages of *S. rubra*, *S. europaea*, *S. persica*, and related taxa (Fig. 6).

## 4 Discussion

Genetic diversity is fundamental to conserving plant species, particularly those inhabiting saline and marginal environments. Moreover, documenting genetic variability lays the groundwork for selecting and propagating genotypes with superior traits, such as high biomass productivity and resilience under saline irrigation, which are critical for potential forage applications. It supports long-term resilience, facilitates local adaptation, and ensures the sustainability of genetic resources in the face of climate change and human-induced pressures.^21^

In this study, we evaluated the genetic variation of *Salicornia iranica* subsp. *sinus-persica*, a morphologically and genetically distinct subspecies^2^ native to coastal areas such as Musa Bay in Iran. Eight primers produced reliable amplification, and the resulting polymorphic profiles revealed clear genetic differentiation, especially between accessions collected from petrochemical-influenced sites and those from natural saline habitats. However, given the limited number of primers and the modest sample size analyzed, these patterns should be regarded as preliminary. Future studies incorporating broader sampling, additional accessions, and complementary molecular markers are recommended to confirm and extend these findings.

This pattern suggests that environmental heterogeneity and restricted gene flow may play important roles in shaping fine-scale genetic differentiation. Notably, Sal-6 formed an isolated cluster, while Sal-1, another genetically divergent accession, was sampled from a coastal site. These patterns align with findings in *Salicornia* populations around Urmia Lake, where high selfing rates and habitat fragmentation promoted local differentiation despite close geographic proximity.^22^ Similarly, studies on arid-adapted chenopods such as annual *Salsola* species have shown that habitat type and environmental stress, rather than geographic distance, significantly influence genetic diversity and population structure.^23^

Sequencing of the ITS region revealed five haplotypes (Table 3), three of which (H3, H4, and a distinct variant of H2) were found exclusively in accessions from Petrochemical sites No. 1 and 2 (Fig. 5). The presence of such private haplotypes may reflect localized divergence resulting from founder effects, limited seed or pollen dispersal, and adaptation to novel environmental conditions. This interpretation is consistent with findings in *S. ramosissima*, where populations inhabiting recently formed anthropogenic saline habitats exhibited distinct genetic structuring. Krüger et al. (2002) suggested that rapid colonization, selfing, and strong site-specific selection can preserve or even generate genetic diversity in secondary habitats, despite potential bottlenecks associated with recent establishment.^24^ A similar pattern has also been observed in grassland species such as *Festuca ovina*, where levels of allozyme polymorphism were high and strongly correlated with environmental factors like soil moisture, pH, and depth.^25^ In that study, even modest changes in habitat conditions over a single decade led to significant shifts in allele frequencies, illustrating the potential for fast and fine-scale genetic responses to environmental gradients.

Such evidence reinforces the idea that subtle ecological variation in saline environments like Musa Bay may exert selective pressures sufficient to drive haplotype-level differentiation in *Salicornia*. In this context, it is notable that haplotype H5 was unique to Sal-1, a coastal accession, suggesting divergence despite the apparent ecological continuity of shoreline habitats. The diploid lineages of *Salicornia* also show swift and effective range expansion, resulting in both widespread and multiple genotypes within small areas.^3^ This pattern underscores the role of cryptic environmental gradients, such as micro-salinity variation, tidal regimes, or localized human impact, as subtle yet effective isolating factors. Such divergence is further reinforced in diploid *Salicornia* by mechanisms like self-pollination, cleistogamy, and restricted dispersal^3^ facilitating lineage separation even in the absence of overt geographic barriers.

Although the ITS region captured notable intraspecific variation in the form of five haplotypes (Fig. 5), phylogenetic analysis failed to recover a fully resolved clade for *S. iranica* subsp. *sinus-persica*. This incongruence between sequence diversity and tree topology is not unexpected, and may be attributed to ancestral polymorphism, recent speciation events, and incomplete lineage sorting. The slow evolutionary rate of the ITS region, along with recent speciation events and incomplete lineage sorting, can obscure species-level relationships.^26^, ^27^, ^28^ Papini et al. (2004), for instance, showed that morphologically distinct tetraploid taxa such as *S. veneta*, *S. emerici*, and *S. dolichostachya* share nearly identical ITS sequences, likely due to their recent divergence and partial reproductive isolation.^26^ Similarly, Murakeözy et al. (2007) found that phylogenetic relationships among *Salicornia* species remain poorly resolved, with sequence differences often uncorrelated to taxonomy or geography.^27^ This pattern is consistent with the evolutionary dynamics of diploid *Salicornia* lineages, where rapid range expansion and localized reproductive isolation, often driven by inbreeding and geographic fragmentation, can generate numerous weakly differentiated genotypes.^2^, ^3^

Notably, BLAST comparisons of the ITS sequences showed high identity to other *Salicornia* species: haplotypes H1–H3 showed > 99 % identity to *S. persica* (EF453460.1), while haplotypes H4 and H5 aligned most closely with *S. europaea* (AB537514.1). Despite these molecular similarities, the studied accessions were morphologically identified as *S. iranica* subsp. *sinus-persica* based on the taxonomic key and diagnostic traits described by Chatrenoor and Akhani (2021). In particular, one key diagnostic criterion is ploidy level: *S. iranica* is diploid (2n = 18), whereas *S. persica* is tetraploid (2n = 36). This cytogenetic distinction supports the current taxonomic treatment, despite the high ITS sequence identity between the taxa.

In the NJ phylogenetic tree, the five ITS haplotypes of *S. iranica* subsp*. sinus-persica* were scattered across several branches rather than forming a monophyletic clade. Haplotypes H2 and H3 clustered together and were positioned adjacent to H4, which in turn was closely associated with H5. Notably, H5 grouped near *S. ramosissima*, while H1 was placed separately but neighbored a clade containing *S. europaea* and *S. rubra*, which themselves were located near *S. persica*. This topology suggests shallow divergence among haplotypes and possible ancestral polymorphism. Moreover, broader phylogenetic analyses based on ETS data have similarly shown *S. iranica* nested within the *S. persica* clade despite morphological and cytogenetic differences.^4^ The incongruent positions of ITS haplotypes in our tree may reflect incomplete lineage sorting, ancestral polymorphism, or past introgression events highlighting the complexity of species delimitation in Salicornia.

Although ITS sequences showed haplotype variation among the accessions of *S. iranica* subsp. *sinus-persica*, no such variation was detected in the *trnH-psbA* plastid region. This contrast can be attributed to the generally conserved nature of chloroplast barcodes, which are characterized by low nucleotide substitution rates and often exhibit limited resolution in distinguishing closely related taxa.^5^ Moreover, their maternal mode of inheritance further restricts their effectiveness for subspecific or species-level delimitation.^29^ Consistent with our observations, the phylogenetic tree presented by Chatrenoor and Akhani (2021), based on combined plastid markers (*atpB-rbcL* and *rpl16*), showed a polytomous grouping of *S. iranica* subsp. *sinus-persica* accessions, indicating unresolved relationships among closely related taxa. Furthermore, our *trnH-psbA* sequences exhibited 100 % identity with *S. persica* in BLAST analysis. However, this high similarity also underscores the limited discriminatory power of this plastid marker for resolving recent divergence events in *Salicornia*.

Importantly, this study provides the first global report of ITS and *trnH-psbA* haplotypes for *S. iranica* subsp. *sinus-persica*, contributing valuable molecular data for future phylogenetic and conservation research. Chatrenoor and Akhani (2021) highlight that such narrowly distributed *Salicornia* taxa represent significant evolutionary units inhabiting vulnerable habitats that are increasingly threatened by anthropogenic activities and climate change.

Overall, the combination of dominant (SCoT) and sequence-based (ITS) markers provided complementary insights into the genetic architecture of *S. iranica* subsp. *sinus-persica*. Identifying distinct haplotypes in both petrochemical and shoreline sites underscores the influence of ecological fragmentation and anthropogenic pressures. As industrial development continues along the Iranian coast, conservation strategies should focus on genetically unique accessions, particularly those in ecologically sensitive microhabitats, to safeguard the evolutionary potential of this endemic halophyte.

It should be emphasized that these findings are preliminary and based on a limited set of molecular markers and accessions. Future research incorporating higher-resolution genomic approaches and broader sampling is needed to validate and refine these patterns.

## 5 Conclusion

This study provides the first molecular characterization of *S. iranica* subsp. *sinus-persica* in Musa Bay, highlighting both genome-wide and sequence-based genetic diversity. Despite the narrow geographic scope, we detected notable genetic diversity, with signs of localized divergence in petrochemical and coastal microhabitats. The haplotype data generated here offer a foundation for future taxonomic and conservation efforts. In light of ongoing industrial development along the Iranian coastline, preserving genetically distinct accessions from ecologically sensitive sites should be a conservation priority. Taken together, these findings advance our understanding of the genetic structure of this halophyte and underscore the importance of conserving genetically distinct accessions as a precautionary measure to safeguard its evolutionary potential.

## CRediT authorship contribution statement

**Fatemeh Nasernakhaei:** Writing – review & editing, Writing – original draft, Supervision, Project administration, Conceptualization. **Mahyar Zahraei:** Writing – original draft, Resources, Investigation, Formal analysis.

## Declaration of competing interest

The authors declare that they have no known competing financial interests or personal relationships that could have appeared to influence the work reported in this paper.
