# Supplementary material for: Genetic differentiation and conservation insights into Salicornia iranica subsp. sinus-persica from Musa Bay using SCoT markers and DNA barcodes
Source: J Genet Eng Biotechnol. 2025 Sep 3;23(4):100563. doi: 10.1016/j.jgeb.2025.100563 (PMC12445711; doi:10.1016/j.jgeb.2025.100563)
Supplement: Supplementary Data 1 [file mmc1.docx]

| **Table S1:** ITS sequences of *Salicornia* species retrieved from GenBank and included in the phylogenetic analysis. | | |
| --- | --- | --- |
| **No.** | **Species Name** | **GenBank Accession Number** |
| 1 | *Salicornia persica* | EF453460.1 |
| 2 | *Salicornia perennans* | AY489249.1 |
| 3 | *Salicornia europaea* | AB537514.1 |
| 4 | *Salicornia europaea* | AY489247.1 |
| 5 | *Salicornia patula* | EF455625.1 |
| 6 | *Salicornia patula* | AJ578058.1 |
| 7 | *Salicornia europaea* | AY181879.1 |
| 8 | *Salicornia europaea* | MT923358.1 |
| 9 | *Salicornia brachystachya* | AY996225.1 |
| 10 | *Salicornia prostrata* | AY489251.1 |
| 11 | *Salicornia ramosissima* | ON685420.1 |
| 12 | *Salicornia pachystachya* | DQ340155.1 |
| 13 | *Salicornia rubra* | OM445472.1 |
| 14 | *Salicornia maritima* | OM445473.1 |
| 15 | *Salicornia europaea* | MF063455.1 |
| 16 | *Salicornia disarticulata* | AY996229.1 |
| 17 | *Salicornia x marshallii* | AY996245.1 |
| 18 | *Salicornia ramosissima* | AY996254.1 |
| 19 | *Salicornia patula* | AJ578059.1 |
| 20 | *Salicornia ramosissima* | AY996251.1 |
| 21 | *Salicornia sp. Akhani* | DQ499343.1 |
| 22 | *Salicornia europaea* | AY181880.1 |
| 23 | *Salicornia ramosissima* | AY996253.1 |
| 24 | *Salicornia patula* | KP114347.1 |
| 25 | *Salicornia patula* | KP114353.1 |
| 26 | *Salicornia patula* | KP114355.1 |
| 27 | *Salicornia ramosissima* | AY996252.1 |
| 28 | *Salicornia europaea* | MF063456.1 |
| 29 | *Salicornia patula* | KP114357.1 |
| 30 | *Salicornia patula* | KP114354.1 |
| 31 | *Salicornia patula* | KP114349.1 |
| 32 | *Salicornia brachiata* | OR826158.1 |
| 33 | *Salicornia patula* | KP114356.1 |
| 34 | *Salicornia emerici* | AJ578055.1 |
| 35 | *Salicornia meyeriana* | AY489248.1 |
| 36 | *Salicornia emerici* | EF455624.1 |
| 37 | *Salicornia patula* | KP114348.1 |
| 38 | *Salicornia fragilis* | AY996240.1 |
| 39 | *Salicornia fragilis* | AY996242.1 |
| 40 | *Salicornia pojarkovae* | DQ340158.1 |
| 41 | *Salicornia procumbens* | AY489250.1 |
| 42 | *Salicornia fragilis* | AY996239.1 |
| 43 | *Salicornia patula* | KP114358.1 |
| 44 | *Salicornia perennans* subsp*. altaica* | KU975215.1 |
| 45 | *Salicornia perennans* | KU975210.1 |
| 46 | *Salicornia emerici* | OL744676.1 |
| 47 | *Salicornia perennans* | KU975209.1 |
| 48 | *Salicornia europaea* | MF063453.1 |
| 49 | *Salicornia europaea* | OQ062633.1 |
| 50 | *Salicornia bigelovii* | EU682686.1 |
| 51 | *Salicornia emerici* | OL744677.1 |
